# Data on the origin, course and distribution of the artery to the human atrioventricular node

**DOI:** 10.1016/j.dib.2018.08.161

**Published:** 2018-08-31

**Authors:** Tomokazu Kawashima, Fumi Sato

**Affiliations:** Department of Anatomy, School of Medicine, Toho University, Tokyo, Japan

## Abstract

This article presents data on the anatomical variation of the origin, course and distribution of the artery to the atrioventricular node in humans. The findings hold clinical significance for coronary intervention, coronary angiography and cardiac pathology in cases of sudden cardiac death. For further interpretation and discussion, the original research article ‘Clarifying the anatomy of the atrioventricular node artery’ by Kawashima and Sato (2018) can be referred [1].

**Specifications table**

**Table:** 

Subject area	*Cardiology/Cardiovascular science*
More specific subject area	*Cardiac anatomy*
Type of data	*Figures*
How data was acquired	*Anatomical data acquisition: dissection of embalmed human hearts under a surgical microscope (OME 5000, Olympus, Japan); histological data acquisition: digital microscope (Olympus BX51, Olympus, Japan)*
Data format	*Raw and analysed*
Experimental factors	*Embalmed samples from donated Japanese cadavers*
Experimental features	*Morphological assessment of the arteries at sub-macroscopic anatomical and histological levels.*
Data source location	*Tokyo, Japan*
Data accessibility	*Data is available with this article.*

**Value of the data**

• The atrioventricular node artery is the only distribution-confirmed artery entering the compact atrioventricular node and/or its nodal extensions.
• The data enhance our understanding of the multiple origins and course of the artioventricular node artery within the inferior pyramidal space.
• The data offer detailed findings and reveals that the artery primarily supplies the proximal part of the atrioventricular conduction axis.

## 1 Data

### 1.1 Origin and course of the atrioventricular node artery

**Fig. 1:**
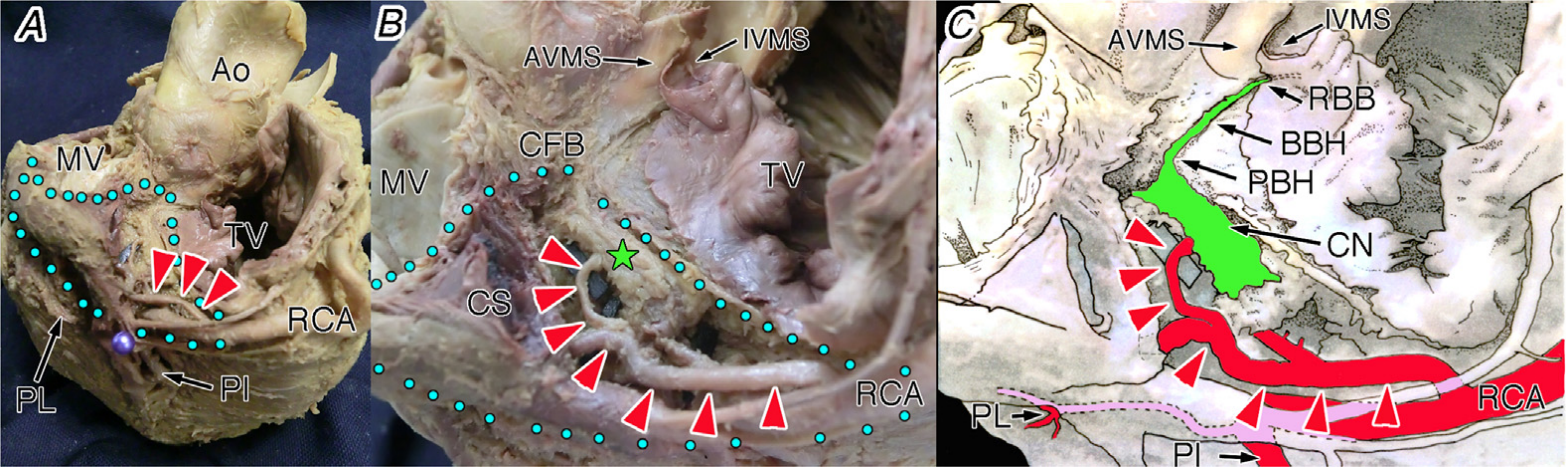
The atrioventricular node artery originating from the #3 coronary segment of the right coronary artery in the ventricles of the heart viewed from the approximately posterior (oesophagus) aspect (A), its magnification (B) and illustration (C). Red arrowheads, the atrioventricular node artery; green star, compact node; and blue dot area, inferior pyramidal space. Ao, aorta; BBH, branching bundle of His; AVMS, atrioventricular part of the membranous septum; CFB, central fibrous body; CN, compact node of the atrioventricular node; CS, coronary sinus; IVMS, interventricular part of the membranous septum; MV, mitral valve; PBH, penetrating bundle of His; PI, posterior interventricular artery; PL, posterolateral branch of the right coronary artery (PL); RBB, right branching bundle; RCA, right coronary artery; TV, tricuspid valve.

**Fig. 2:**
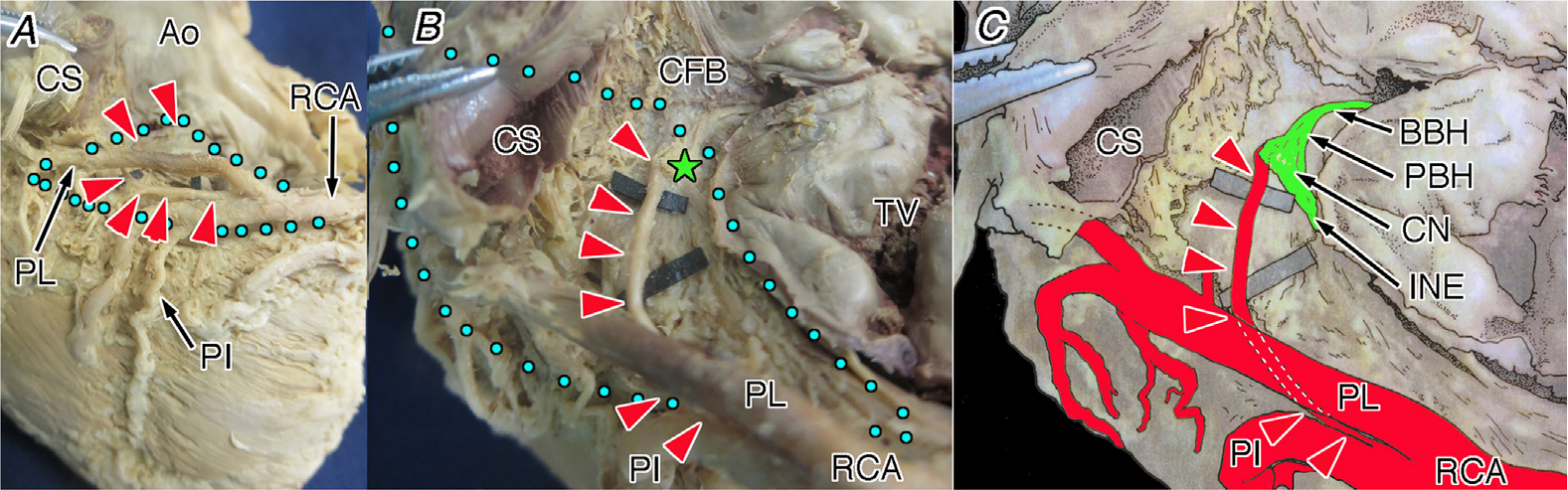
The atrioventricular node artery originating from the posterior interventricular branch (#4PI) of the right coronary artery in the whole heart viewed from the approximately inferior (diaphragmatic) aspect (A), its magnification (B) and illustration (C). Red arrowheads, the atrioventricular node artery; green star, compact node; and blue dot area, inferior pyramidal space. Ao, aorta; BBH, branching bundle of His; CFB, central fibrous body; CN, compact node of the atrioventricular node; CS, coronary sinus; INE, inferior nodal extension; PBH, penetrating bundle of His; PI, posterior interventricular artery; PL, posterolateral branch of the right coronary artery (PL); RCA, right coronary artery; TV, tricuspid valve.

**Fig. 3:**
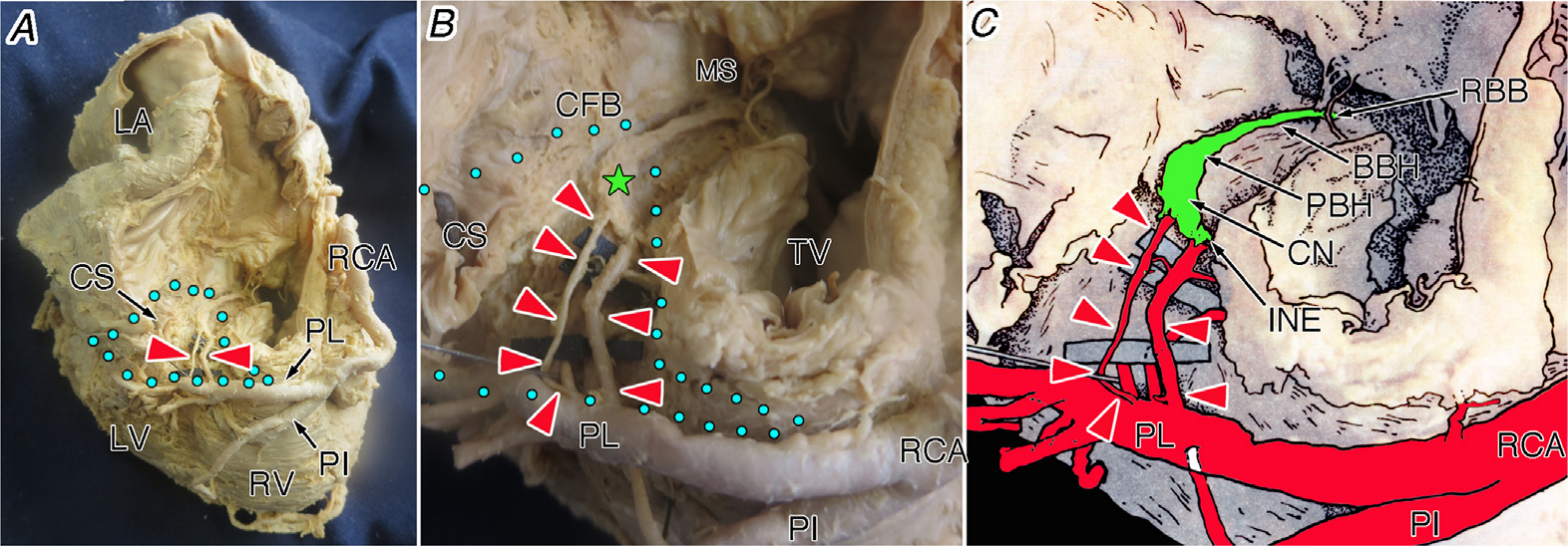
The atrioventricular node artery originating from the proximal posterolateral branch (proximal #4PL) of the right coronary artery in the whole heart viewed from the approximately inferior (diaphragmatic) aspect (A), its magnification (B) and illustration (C). Red arrowheads, the atrioventricular node artery; green star, compact node; and blue dot area, inferior pyramidal space. BBH, branching bundle of His; CFB, central fibrous body; CN, compact node of the atrioventricular node; CS, coronary sinus; INE, inferior nodal extension; MS, membranous septum; PBH, penetrating bundle of His; PI, posterior interventricular artery; PL, posterolateral branch of the right coronary artery (PL); RBB, right branching bundle; RCA, right coronary artery; TV, tricuspid valve.

**Fig. 4:**
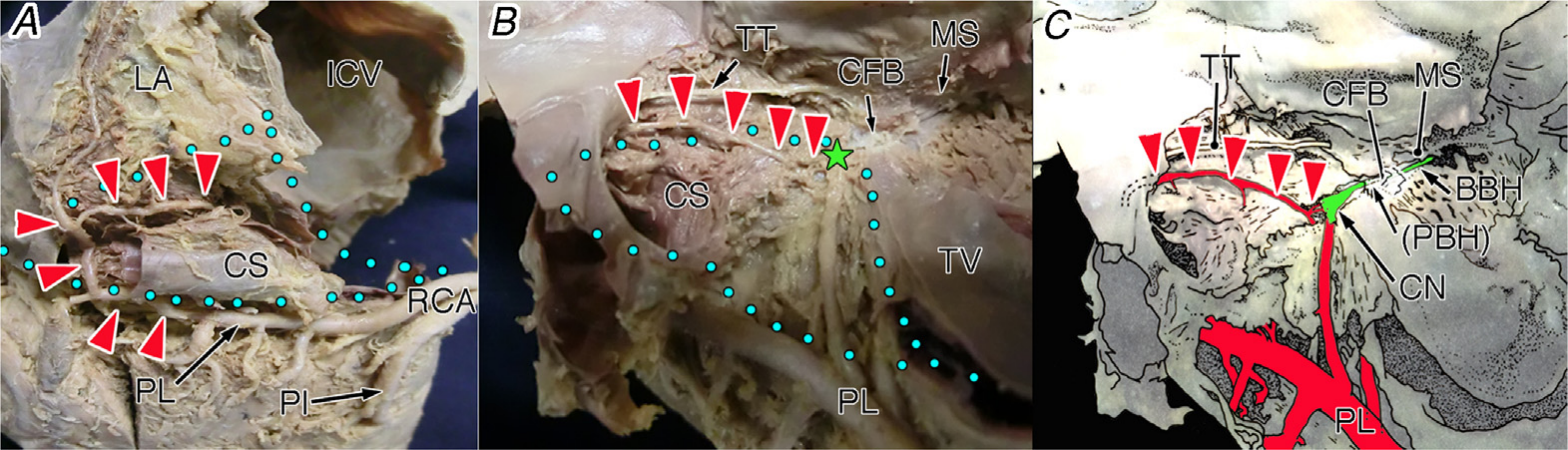
The atrioventricular node artery originating from the distal posterolateral branch (distal #4PL) of the right coronary artery and coursing laterally to the coronary sinus in the whole heart viewed from the approximately inferior (diaphragmatic) aspect (A), its inner view of right chamber (B) and illustration (C). Red arrowheads, the atrioventricular node artery; green star, compact node; and blue dot area, inferior pyramidal space. BBH, branching bundle of His; CFB, central fibrous body; CN, compact node of the atrioventricular node; CS, coronary sinus; ICV, inferior caval vein; MS, membranous septum; PBH, penetrating bundle of His; PI, posterior interventricular artery; PL, posterolateral branch of the right coronary artery (PL); RCA, right coronary artery; LA, left atrium; TT, tendon of Todaro; TV, tricuspid valve.

**Fig. 5:**
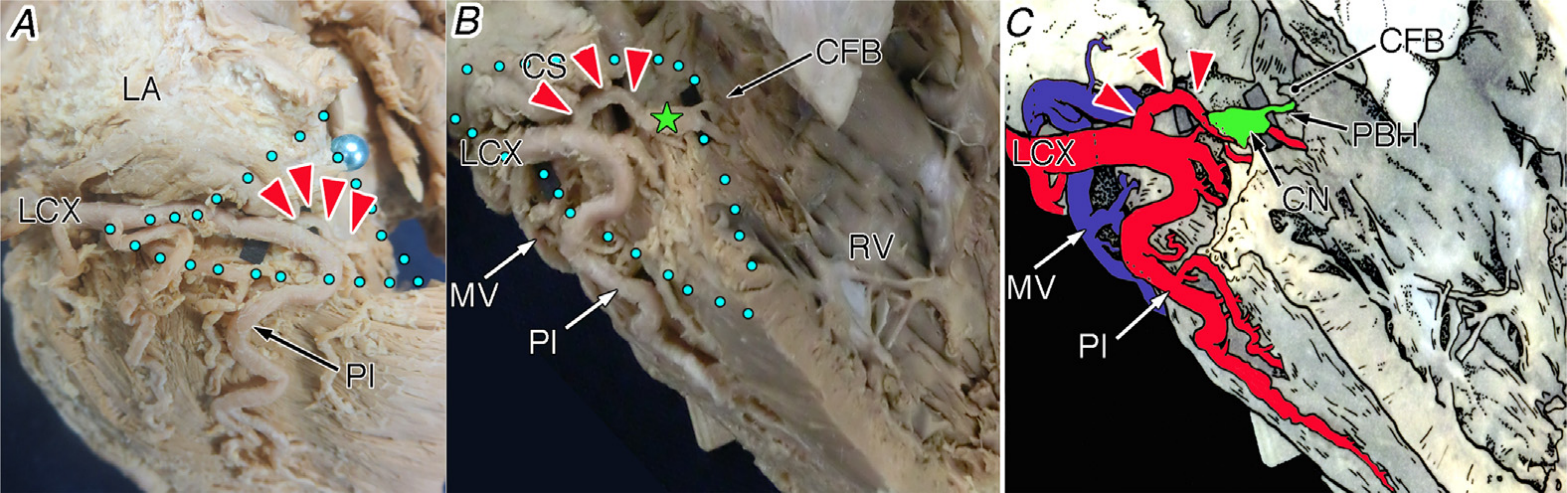
The atrioventricular node artery originating from the left circumflex branch (#13) of the left coronary artery in the whole heart viewed from the approximately inferior (diaphragmatic) aspect (A), its magnification of right chamber (B) and illustration (C). Red arrowheads, the atrioventricular node artery; green star, compact node; and blue dot area, inferior pyramidal space. CFB, central fibrous body; CN, compact node of the atrioventricular node; CS, coronary sinus; LA, left atrium; LCX, left circumflex branch of the left coronary artery; MV, middle cardiac vein; PBH, penetrating bundle of His; PI, posterior interventricular artery; RV, right ventricle.

Using our specialised dissection technique of the atrioventricular conduction axis based on the histological criteria [1], [2], [3], [4], 164 arterial branches (average, 1.6 branches) to the compact atrioventricular node and its nodal extensions were found in 101 of 103 cases (98.1%). In the pyramidal space, we found five origins of the atrioventricular node artery were in the modified AHA coronary guideline, which are as follow: distal RCA [#3, 17/164 branches (10.4%); Fig. 1]; right posterior interventricular artery [#4PI, 12/164 branches (7.3%); Fig. 2]; proximal RCA posterolateral branch [proximal #4PL, 126/164 branches (76.8%); Fig. 3]; distal RCA posterolateral branch [distal #4PL, 3/164 branches (1.8%); Fig. 4]; distal left circumflex artery [#13, 6/164 branches (3.7%); Fig. 5].

Only the distal #4PL-type courses detoured the coronary sinus clockwise, whereas the other four types ascended within the IPS and distributed to the atrioventricular node.

### 1.2 Distribution of the atrioventricular node artery

**Fig. 6:**
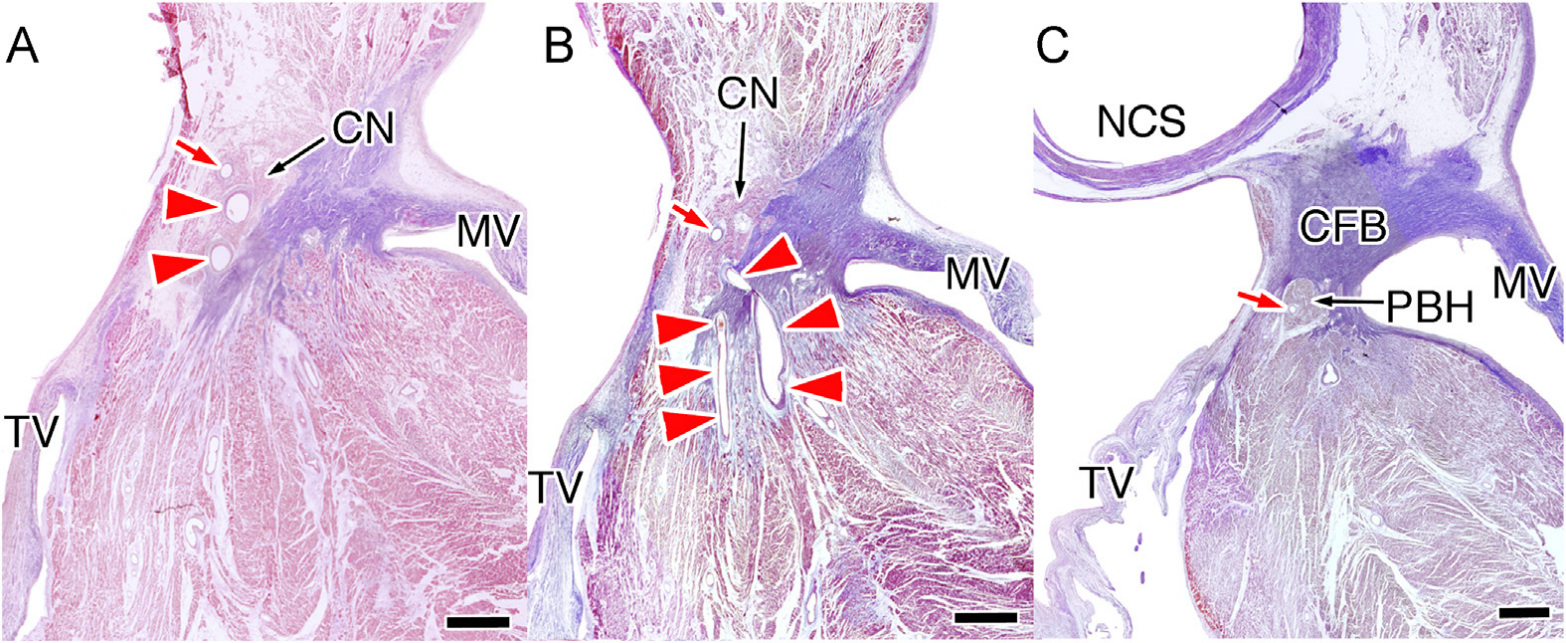
Photographs showing that the atrioventricular node artery supplies the proximal part of the atrioventricular conduction axis. In three penetrating atrioventricular node arteries within the compact node (A), two thick arteries (shown in red arrowheads) supplies to and leaves at the distal compact node (B), and only one small artery (shown in red arrow) runs within the penetrating branch of His (C). CFB, central fibrous body; CN, compact node; MV, mitral valve; NCS, non-coronary aortic sinus; TV, tricuspid valve.

We examined the serial sections of the atrioventricular junctions. The penetrating arterial branches of the compact atrioventricular node supplied the atrioventricular conduction axis up to the distal compact node (23/39 branches, 71.8%, Fig. 6), the penetrating bundle of His (6/39 branches, 15.4%) and the branching bundle of His (5/39 branches, 12.8%).

## 2 Experimental design, materials and methods

### 2.1 Specimens

We used a total of 120 human hearts obtained from human cadavers for data acquisition (range, 49–105 years; average 85.4±10.6 years). The specimens were fixed using 10% formalin and preserved in 30% alcohol for >1 year. We used 103 hearts for the sub-macroscopic anatomic examination of the atrioventricular node artery. The remaining 17 hearts were used for serial sections at 20-μm intervals. Further, we excluded any heart-related abnormal conditions from this data.

### 2.2 Dissection

We used attitudinally correct terminologies were used in this study [5], [6], [7].

We dissected 103 embalmed human hearts using forceps for optic surgery (Dumont #4; World Precision Instruments, USA) under a binocular microscope designed for neurological surgery (*OME 5000*, Olympus, Tokyo, Japan). We recorded all dissection images with a digital camera (*IXY digital 800IS*; Canon, Japan).

### 2.3 Histology

We conducted histological reconfirmation of the exposed atrioventricular conduction axis and comprehensively examined the destination of the artery to the atrioventricular node using Masson׳s trichrome stain. In addition, we examined 17 hearts to determine the destination of the atrioventricular node artery. Furthermore, we acquired histological data using a digital microscope (*Olympus BX51; Olympus, Japan*).
