# Supplementary material for: Data on the origin, course and distribution of the artery to the human atrioventricular node
Source: Data Brief. 2018 Aug 31;20:1057–61. doi: 10.1016/j.dib.2018.08.161 (PMC6140359; doi:10.1016/j.dib.2018.08.161)
Supplement: Supplementary file 1 — Supporting information [file mmc1.docx]

**Conflict of interest**

The authors declare that they have no competing interests.
